# Tourniquet use in different emergency medicine professional groups, results from a regional cross-sectional online survey

**DOI:** 10.1186/s12873-026-01674-w

**Published:** 2026-08-03

**Authors:** P. Hilbert-Carius, H. Wrigge, K. zur Nieden, B. Stichert, A. Großstück, Michael Lautenschläger

**Affiliations:** 1 Department of Anaesthesiology, Intensive Care and Emergency Medicine, Pain Therapy Bergmannstrost Hospital Halle (Saale), Halle (Saale), Germany; 2 International Aid Group e.V. Leipzig, Leipzig, Germany; 3 DRF Luftrettung Halle / Oppin, Halle (Saale), Germany; 4 Medical Faculty, Friedrich Schiller University, Jena, Germany; 5 ADAC-Luftrettung Dölzig, Dölzig, Germany; 6 Medical Faculty, Martin-Luther-University Halle, Halle (Saale), Germany; 7 Emergency Medical Services in Halle (Saale) and Northern Saale District, Halle (Saale), Germany

**Keywords:** Haemorrhage, Extremity trauma, Pre-hospital, Emergency medical service personnel, Pre-hospital physicians

## Abstract

**Background:**

Traumatic haemorrhage is one of the most common causes of death in patients with injuries. Targeted pre-hospital haemostasis can reduce morbidity and mortality. The use of tourniquets is advisable for external bleeding in the extremities that cannot be treated with compression or wound packing. There are recommendations for the use of tourniquets. Little is currently known about the level of training and frequency of use of various professional groups of potential tourniquet users.

**Methods:**

A regional cross-sectional online survey was conducted on the level of training, frequency of use, and adherence to guidelines for tourniquets among various pre-hospital professional groups (emergency medical technician– EMT, advanced emergency medical technician–AEMT, Paramedical, Helicopter Emergency Medical Services-Technical Crew member–HEMS-TC, pre-hospital physician), to get a regional convenience sample. To obtain an overview of the level of training and use of tourniquets among various professional groups, we conducted a regional online survey on ground-based emergency medical services in the region of Halle/Northern Saale District in Saxony-Anhalt, as well as on helicopter emergency medical services in the Halle/Leipzig metropolitan region. The different professional groups were compared with regard to theoretical and practical training, frequency of use, and adherence to the existing guidelines.

**Results:**

178 participants (74% male, 26% female) took part and answered the questionnaire. Among respondents who reported professional group were 47% pre-hospital physicians, 32% paramedics, 9% EMT, 7% HEMS-TCs, 4% AEMT. Statistically significant differences were found in theoretical education, with the lowest rate of education in the EMT group (67%) and the highest rate in the paramedic group (96%). When comparing theoretical education of all the emergency medical service personnel (91%) against the pre-hospital physicians (73%), it was significantly higher in the emergency medical service personnel group. No differences were found in practical training or frequency of use. Physicians had a significant higher adherence to the guidelines for location for applying a tourniquet.

**Conclusion:**

In addition to theoretical education, no statistically significant differences were detected in this regional sample in terms of practical training or frequency of use. With regard to adherence to existing guidelines, physicians had a significantly higher adherence for location for applying a tourniquet. Standardized training and periodic refresher education may help ensure consistent tourniquet knowledge and application across prehospital professional groups.

**Supplementary Information:**

The online version contains supplementary material available at 10.1186/s12873-026-01674-w.

## Background

Traumatic haemorrhage is one of the most common causes of death in patients with injuries [1, 2]. Targeted pre-hospital treatment can reduce morbidity and mortality [3]. On the one hand, limb haemorrhage is often underestimated as a source of bleeding, but on the other hand, it can be effectively treated pre- and in-hospital with pressure bandages, wound packing or tourniquets [4, 5]. The use of tourniquets for limb haemorrhage that cannot be stopped with a pressure bandage or where the tactical situation requires the temporary application of a tourniquet has gained importance owing to positive experiences in the military and the increase in terrorist activities over the last two decades [6–8]. Nevertheless, the majority of experiences and data originate from war and crisis zones (Afghanistan, Iraq, Israel, Ukraine) [8–11]. More recently, data from civilian emergency medicine services (EMS) have also been increasingly reported [12–16]. However, there are only a few publications available from European countries, and little is known about the differences in theoretical and practical training, frequency of use, and adherence to existing guidelines between different professional groups (emergency medical technician/basic emergency medical technicians–EMT, advanced emergency medical technician-AEMT, paramedic, Helicopter Emergency Medical Services-Technical Crew member–HEMS-TC, pre-hospital physician) [6, 17–19]. Within the German EMS system, there are currently several levels of qualification for non-physician staff (“Rettungssanitäter” -EMT, “Rettungsassistent” -AEMT, “Notfallsanitäter” -Paramedic and “Notfallsanitäter” with a special HEMS and critical care education -HEMS-TC). In the future AEMT will disappear as no further training is provided for it.

## Methods

**Fig. 1:**
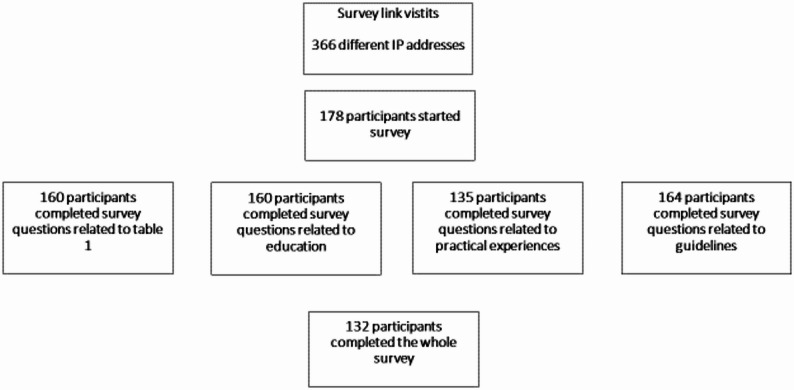
Flow diagram of survey links visits and participants in the different sections analysed

To obtain an overview of the level of training and use of tourniquets among the various professional groups, we conducted a regional online survey in ground-based EMS in the Halle (Saale) / Northern Saale District in Saxony-Anhalt, Germany, as well as for helicopter emergency medical services (HEMS) in the Halle (Saale) /Leipzig metropolitan region. This area comprises 24 EMS stations, including five physician-staffed rapid-response vehicles, two HEMS bases operating four helicopters, and one location with a physician-staffed intensive care transport vehicle. A questionnaire with multiple questions regarding education, availability, level of training, experience in the use of tourniquets in EMS and HEMS, and personal opinions was available online from 02.07.24 to 15.10.2024. A QR code to access the survey was sent to 24 EMS bases and two HEMS bases (including four Helicopters) of the participants via email and was also available in the Emergency department of the hospital. The QR code led to the LamaPoll website of the survey. To avoid duplicate entries IP checks were used. The questionnaire (see subl. Figure 1) was developed by three experienced emergency physicians and evaluated by the author to obtain relevant information regarding the use of tourniquets by pre-hospital emergency medical personnel and emergency doctors in a local-regional area. It consists of various sections (general, training, availability, theoretical and practical aspects, and opinions) with a total of 44 questions. To validate the questionnaire, a brief pilot survey using the questionnaire was carried out at an ambulance station located directly next to our hospital, with the result that three questions were rephrased. In the current study, we analysed questions regarding education/training, practical experience in application, and adherence to guidelines. Guidelines in the context of the survey means the “Stop the bleeding” section of the German Guideline on the Treatment of Patients with Multiple and/or Severe Injuries and the German guidelines on the use of tourniquets issued by the German Society for Anaesthesiology and Intensive Care Medicine even though there are still international recommendations [5, 18, 20, 21]. The questions 17), 18), 19) of the survey (see subl. Figure 1) were addressing specific recommendations of the mentioned guidelines or addressing important points in the text of the guidelines and were analysed to investigate adherence to the guidelines recommendations. We compared the different professional groups against each other where appropriate and all the emergency medical service personnel (EMT, AEMT, Paramedic, and HEMS-TC) against the pre-hospital physicians, if significant differences were found between the professional groups. If not all participants answered every question of the survey, all available answers were analysed. Statistical analysis was mainly descriptive, and differences between groups were compared using Fisher’s exact test for categorical data. Statistical significance was set at a p-value < 0.05. All computations were performed using GraphPad Prism (GraphPad Software, 225 Franklin Street FL 26 Boston, MA 02110, USA).

Data collection was approved by hospital management and was in accordance with the European General Data Protection Regulation. As no personal data that could reveal the identity of a participant were collected as part of the study, the Ethics Committee of the Saxony-Anhalt Medical Association did not consider a new vote necessary and referred to the existing vote (56/21) in a study on tourniquets already conducted by the authors [22].

A general overview of the data collected has already been published in a German-language journal [23]. In the supplemental material a general overview of data already been published in the German-language journal is available.

## Results

During the specified period, the online survey was visited by 366 interested parties from different IP addresses, of whom 178 (49%) among individuals who accessed the survey link ultimately participated in the complex survey. Of the 178 participants (74% male, 26% female), 132 (74%) completed the survey. Unfortunately, we cannot provide information on the total number of clinicians working at the selected EMS and HEMS bases.

The results in the following sections refer to all available responses, not just the 132 participants who completed the whole questionnaire. This means that the number of responses/participants may vary between questions. Therefore, the denominator (number of participants) for each survey item analysed is mentioned in brackets in the sub-headlines as well as in Fig. 1.

Table 1 provides an overview of the available general information such as age, qualifications, type of emergency vehicle, and length of service in the EMS of the participants.

**Table 1:** Age, qualifications, EMS vehicle and length of service in the emergency medical services of the 160 participants who answered the specific questions

Age of participants	20–29 years 17% (*N* = 27)	30–39 years 31% (*N* = 50)	40–49 years 32.5% (*N* = 52)	50–59 years 17% (*N* = 27)	> 60 years 2.5% (*N* = 4)
Profession of participants^1^	physician 47% (*N* = 75)	HEMS-TC 7% (*N* = 11)	paramedic 32% (*N* = 51)	AEMT 4% (*N* = 6)	EMT 9% (*N* = 15)
Emergency vehicle^2^	Ambulance 46% (*N* = 73)	Rapid response vehicle 54% (*N* = 86)	Rescue Helicopter 29% (*N* = 47)	Intensive care transport vehicle 9% (*N* = 14)	More than one vehicle 36% (*N* = 57)
Time in EMS / HEMS^3^	< 1 year 0%	1–2 years 8% (*N* = 13)	3–5 years 22% (*N* = 35)	6–10 years 20% (*N* = 32)	> 10 years 50% (*N* = 80)

^1^Two participant did not specify the profession, ^2^multiple answers possible, ^3^ time since completing training in the emergency medical service

Of the 75 participating physicians, 63 (84%) were consultants (senior doctors) and 12 (16%) residents (junior doctors), which were divided into the following medical specialties: 56 (74,5%) anaesthesiology and intensive care medicine, 8 (10.5%) surgery, 9 (11%) internal medicine, and 3 (4%) others.

### Education and training (160 participants)

91% (*N* = 146) of the respondents had some kind of theoretical education and / or practical training in the tourniquet use, and 9% (*N* = 14) did not, with no statistically significant differences between the professional groups. Theoretical education was completed by 81% (*N* = 130) of the respondents, and practical training by 87% (*N* = 139). The difference in the theoretical education between the professional groups was statistically significant (*p* = 0.0062), with the lowest rate of education in the EMT group 67% (*N* = 10) and the highest rate in the paramedic group 96% (*N* = 49). When comparing the rate of theoretical education between all the emergency medical service personnel 91% (*N* = 77) to the pre-hospital physicians, 73% (*N* = 56), theoretical education was significantly higher in the emergency medical service personnel group (*p* = 0.003; odds ratio 3.9; 95% CI 1.4–11.7). 43% (*N* = 69) of all respondents stated that they had received training or education on their own initiative (EMT 53%, AEMT 33%, paramedics 53%, HEMS-TC 27%, physicians 37%). The differences between the professional groups were not statistically significant. As part of their vocational training in the EMS, 81% (*N* = 69) of the non-physicians received training and a total of 29% (*N* = 25) received training in the form of special courses (PHTLS-Pre-Hospital Trauma Life Support, etc.). 51% (*N* = 38) of the pre-hospital physicians received their training as part of the emergency doctor course and 55% (*N* = 41) in the form of special courses.

### Practical experience in application (135 participants)

57% (*N* = 77) of respondents had applied a tourniquet to at least one patient, and 13% (*N* = 18) have had to apply more than one tourniquet at the same time. There were no statistically significant differences between the professional groups in terms of the necessity of tourniquet application. 80% (*N* = 8) of EMT, 67% (*N* = 4) of AEMT, 55% (*N* = 21) of paramedics, 73% (*N* = 8) of HEMS-TCs, and 54% (*N* = 37) of doctors stated that they had already applied a tourniquet. Regarding the frequency of use, 29% (*N* = 39) of the respondents stated that they had used a tourniquet 1–2 times, 21% (*N* = 29) 3–5 times, 6% (*N* = 8) 6–10 times and 1% (*N* = 1) > 10 times. When comparing the necessity of tourniquet application of all the emergency medical service personnel 67% (*N* = 41) against the pre-hospital physicians 54% (*N* = 37), the difference was not statistically significant.

### Adherence to existing guidelines (164 participants)

The location for applying a tourniquet in the pre-hospital setting was defined by respondents as follows: 77% (*N* = 126) as distally as possible, but sufficiently proximally (at least 5 cm / a hand’s cm/width) from the source of bleeding, 12% (*N* = 20) directly above the site of bleeding, and 11% (*N* = 18) above the joint of the affected limb. There was a statistically significant difference between the professional groups (*p* = 0.016). When comparing pre-hospital physicians against the EMS personnel group physicians had a significant higher adherence to the guidelines for location for applying a tourniquet (*p* = 0.029; odds ratio 4.5, 95% CI 1.5–21.7). Regarding the indication for pre-hospital tourniquet use, 93% of the answers were in accordance with current guidelines, with no statistically significant differences between the professional groups. Considerable differences were observed in the responses regarding maximum occlusion time for the tourniquet. 1.25% (*N* = 2) of respondents estimated the maximum occlusion time for a tourniquet to be 30 min, 16% (*N* = 26) estimated it to be 60 min, 8.5% (*N* = 14) estimated it to be 90 min, the majority 66.5% (*N* = 109) estimated it to be 120 min, 6.5% (*N* = 11) estimated it to be 180 min, and 1.25% (*N* = 2) estimated it to be unlimited. Therefore 92% of answers were within the recommended maximum occlusion time of < = 120 min. No significant differences were found between professional groups. Across the three guideline-related survey items, 90% of selected answer options were classified as guideline-concordant. This does not represent the proportion of respondents who were fully guideline-adherent across all items. Supplemental Table 1 reflects proportion of guideline-concordant answer options, not fully adherent individuals. Supplemental Table 1 also gives a respondent-level composite outcome on guideline adherence.

Another important finding of the survey was that all rescue vehicles (ambulances, rescue helicopters, etc.) were reported to be equipped with tourniquets. This finding should be interpreted as self-reported availability rather than an independently verified inventory but it is in accordance with local regulations for ambulances, rapid response vehicles and rescue helicopters.

## Discussion

Our survey demonstrates that most prehospital personnel reported receiving theoretical and/or practical training in tourniquet use, and more than half reported having previously applied a tourniquet. We included three questions in the questionnaire to assess the key points of the above-mentioned guidelines: the indications for using a tourniquet, the site of application, and the maximum duration of occlusion. According to the guidelines the following is recommended [5, 18]:

Indications:

- Amputation injury proximal to the wrist or foot.
- Multiple bleeding sites on a limb, which together may result in significant blood loss.
- Severe bleeding from a limb accompanied by a critical A, B or C problem.
- Severe bleeding from a limb where the injury is inaccessible (e.g. a trapped person).
- Inability to stop bleeding using pressure dressings or similar methods.
- Management of severe bleeding from a limb in the dark.
- Severe bleeding from limbs in a mass casualty incident.
- Severe bleeding from limbs under time pressure in hazardous situations.

Site of application:

- As far distal as possible – but sufficiently proximal (at least 5 cm / a hand’s width) to the source of bleeding.
- Not over joints.
- Not over wound cavities, foreign bodies or open fractures.
- In hazardous situations, in the dark, in cases of multiple bleeds on a limb, mass casualty incidents, explosion injuries and open fractures, apply the tourniquet as proximally as possible.

Maximum Occlusion time:

 • 120 min (the shorter, the better).

Surveys and descriptive analyses in civilian EMS have already been conducted. Several studies have characterized the patterns, trends, and outcomes of pre-hospital tourniquet use in civilian settings [12, 15, 24, 25].

Large-scale analyses using the US data have described the prevalence, predictors, and demographic characteristics of tourniquet use by civilian EMS, showing an increase in adoption, especially in urban and advanced life support settings. These surveys found that tourniquets are most commonly used for traumatic injuries, with upper extremity injuries being more frequent than lower extremity injuries, and that the prevalence of their use has increased [12].

An American survey of civilian pre-hospital providers revealed significant knowledge gaps and a low number of trained providers [25]. The survey was comparable to our study in terms of the number of participants (*N* = 151) and non-physician professional groups. In contrast to our study, only 17% of the participants had used tourniquets in the pre-hospital setting, but the survey was already more than 13 years old. However, one must bear in mind that tourniquet use in civilian EMS is increasing [25].

Other regional and multicentre studies consistently demonstrate that tourniquet use in civilian EMS is increasing, is effective for haemorrhage control, and is associated with low complication rates when applied appropriately [26–29]. Pre-hospital tourniquet use in EMS is associated with improved survival, reduced shock on arrival, decreased transfusion requirements, and a low rate of serious limb complications [25, 26, 28–32].

In contrast to the US EMS and many other American/South American countries, in the German-speaking zone and some other European countries, pre-hospital physicians can regularly be found in EMS and HEMS. No large-scale published surveys or studies have specifically focused on the use of tourniquets by pre-hospital physicians in EMS. To the best of our knowledge, the current study is the first to investigate the education/training, practical experience, and guideline adherence of tourniquet application by pre-hospital physicians. The lower self-reported rate of theoretical education among hospital physicians suggests a potential gap in onboarding, continuing education, or vehicle-specific equipment familiarization. Basic training and repeated refresher education may help ensure consistent tourniquet knowledge and application across prehospital professional groups.

In line with our results, the literature shows that the level of training in the use of tourniquets among pre-hospital physicians is heterogeneous and often inadequate. Studies have shown that although tourniquets are increasingly being used in civilian EMS, the correct indications and applications are often suboptimal. Tourniquets are regularly applied without clear indication or are placed incorrectly, which points to deficits in education and training [33–35].

## Limitations

The study is a regional convenience sample with unknown response rate and unknown eligible denominator, all measures are self-reported and limited generalizability. The study may be subject to the following biases: selection bias, self-report bias, and recall bias regarding previous tourniquet use. Furthermore, the study reveals the following shortcomings: inability to calculate a true response rate, no objective assessment of correct tourniquet application, no clinical patient-level outcomes, potential overlap with prior publication and a not formally validated questionnaire. The high number of physicians in this study is explained by the fact that, for example, at HEMS bases the ratio of HEMS-TC / Paramedic to pre-hospital physicians is approximately 1 to 3, and at rapid-response vehicles it is approximately 1 to 5. Nevertheless, a certain kind of response bias cannot be totally ruled out. The overall number of participants is small therefore subgroup analyses are underpowered. The number of participants of some EMS professional groups is quite low and therefore makes the results appear to be based more on association than on clear evidence. Given the small number of participants in some groups, it is possible that results which are currently not statistically significant might become significant if the sample size were larger.

## Conclusion

This regional cross-sectional online survey suggests that most prehospital personnel reported receiving theoretical and/or practical training in tourniquet use, and more than half reported having previously applied a tourniquet. Prehospital physicians reported lower rates of theoretical education than non-physician EMS personnel, but showed higher guideline-concordant responses regarding tourniquet placement. Because the study used a regional convenience sample with an unknown eligible denominator, self-reported measures, and small subgroup sizes, the findings should be interpreted as exploratory. Standardized training and periodic refresher education may help ensure consistent tourniquet knowledge and application across prehospital professional groups.

## Supplementary Information

Below is the link to the electronic supplementary material.

Supplementary Material 1

Supplementary Material 2

## Data Availability

The datasets used and analysed during the current study are available from the corresponding author on reasonable request.

## Abbreviations

AEMT: Advanced emergency medical technician / intermediate emergency medical technicians
EMS: Emergency medical service
EMT: Emergency medical technician / basic emergency medical technicians
HEMS: Helicopter emergency medical service
HEMS-TC: Helicopter emergency medical services - technical crew member
PHTLS: Pre-hospital trauma life support
US: United States
