# Supplementary material for: Tourniquet use in different emergency medicine professional groups, results from a regional cross-sectional online survey
Source: BMC Emerg Med. 2026 Aug 3;26:214. doi: 10.1186/s12873-026-01674-w (PMC13445843; doi:10.1186/s12873-026-01674-w)
Supplement: Supplementary file 1 [file 12873_2026_1674_MOESM1_ESM.docx]

**Survey on the level of training and experience in the use of tourniquets in ground-based and air ambulance services**

Dear Colleague,

The aim of this survey is to find out more about the level of training and the frequency of use of tourniquets in the emergency medical services.

The results of the analysis will be used to provide further information on the use of tourniquets, safety in their handling, any training needs and problems encountered.

The survey will take approximately 20 minutes to complete.

The survey is anonymous; it is not possible to link your contact details to your answers. The evaluator will only carry out an overall analysis, without any possibility of identifying individual questionnaires.

If you have any questions regarding the survey, please contact Michael Lautenschläger (michael.lautenschlaeger@bergmannstrost.de).

Only if many colleagues complete the questionnaires can a meaningful analysis be produced and improvements implemented!

We look forward to a high level of participation and thank you for your support.

With best regards,

Michael Lautenschläger

**General section**

Single-choice question*

1) **Please select your gender**

Male

Female

Diverse

Prefer not to say

Single-choice question*

2) **Please select the age category that applies to you**

< 30 years

30–39 years

40–49 years

50–59 years

> 60 years

Single-choice question*

3) **Please state your occupational group**

Paramedic

Emergency Medical Technician

Advanced Emergency Medical Technician

HEMS-TC

Doctor

No information provided

Single-choice question

4) **If you selected ‘Doctor’ in the previous question, please state your level of training**

Doctor in further training

Specialist

Single-choice question

5) **If you selected “Doctor” in question 4, please state your specialism or, for doctors in training, your intended specialism**

Anaesthesiology

Surgery

Internal medicine

Other

Multiple-choice question*

6) **Which emergency service do you work for?** (If you work for more than one emergency service, please complete the questionnaire only once and answer the questions only for the emergency service you are currently working for.)

Ambulance

Rapid Response Vehicle

Rescue helicopter/critical care transport helicopter (HEMS)

Ambulance for inter-hospital transfer

Single-choice question*

**7) How long have you been working in the emergency medical services?**

1–2 years

3–5 years

6–10 years

>10 years

**Training section**

Single-choice question*

8) **Have you already received training in the use of a tourniquet?**

Yes

No

No information provided

Single-choice question*

9) **Have you received theoretical training in the use of a tourniquet (e.g. instructional video)?**

Yes

No

No information provided

Single-choice question*

10) **Have you received practical training in the use of a tourniquet** (e.g. independent use of different types of tourniquets)**?**

Yes

No

No information provided

Multiple-choice question*

11) **In what context did training (theoretical or practical) in the use of a tourniquet take place?**

On your own initiative

Training as a paramedic / emergency medical technician / HEMS

Emergency doctor course

Advanced course formats such as PHTLS/ATLS/TTTS

No information provided

**Availability**

Single-choice question*

12) **Does your emergency vehicle have at least one tourniquet?**

Yes

No

No information provided

Please provide an answer

13) **If yes, please specify the system available**

…………

Single-choice question*

14) **Does your ambulance / emergency vehicle have other haemostatic equipment, e.g. haemostatic agents?**

Yes

No

No information provided

Please provide an answer*

15) **How many tourniquets does your current ambulance / emergency vehicle have?**

……

**Theoretical section**

Single-choice question*

16) **How long do you estimate it takes to apply a tourniquet?**

< 1 min

1–3 min

> 3 min

Multiple-choice question*

17) **When would you apply a tourniquet?**

for injuries to limbs that can be compressed effectively

regardless of the effectiveness of a pressure bandage

after applying a pressure bandage, but if bleeding persists

for multiple injuries to a limb, with several sources of bleeding

in the event of multiple casualties

for inaccessible limb injuries

for limb injuries close to the trunk

to stop bleeding whilst other actions are required, e.g. securing the airway at the same time

only as a last resort

Multiple-choice question*

18) **Where would you place a tourniquet?**

directly above the site of bleeding

as distally as possible on the limb, but sufficiently proximal to the source of bleeding (e.g. a hand’s width)

directly over the bleeding wound (for better compression)

over a possible fracture (to stabilise the fracture)

over the joint of the affected limb

Single-choice question*

19) **How long do you estimate the maximum duration of a tourniquet to be?**

30 min

60 min

90 min

120 min

150 min

180 min

unlimited

**Practical section**

Single-choice question*

20) **Have you ever applied a tourniquet?**

Yes

No

No information provided

Single-choice question*

21) **How many times have you applied a tourniquet?**

1 - 2

3 - 5

6 - 10

>10

None

No information provided

Single-choice question*

22) **Have you ever had to apply more than one tourniquet at the same time?**

Yes

No

No information provided

Single-choice question*

23) **Do you administer analgesia when applying a tourniquet?**

Non-opioid

Opioid

Ketamine/Ketanest

Combination

No analgesics

No information provided

Multiple-choice question*

24) **Which substances do you use for analgesia when applying a tourniquet?**

Paracetamol

Metamizole

Piritramide

Fentanyl

Sufentanil

Ketamine — Ketanest

Other

None

Single-choice question*

25) **Have patients reported symptoms such as pain or paraesthesia despite analgesia or analog -sedation?**

Yes

No

No information provided

Single-choice question*

26) **Do you record the time when applying a tourniquet?**

Yes

No

No information provided

Multiple-choice question*

27) **Were further intensive care measures required during the application of the tourniquet?**

Airway management/artificial ventilation

Analgesic sedation

Insertion of a chest drain

CPR

Other

Single-choice question*

28) **Did the application of a tourniquet achieve sufficient haemostasis that could not previously be achieved with other measures?**

Yes

No

Not always

No information provided

Single-choice question*

29) **Were other haemostatic measures attempted before applying a tourniquet?**

Yes

No

No information provided

Single-choice question*

30) **Under controlled conditions and with adequate haemostatic measures in addition to the tourniquet (e.g. pressure dressing and haemostatic agents), would you consider converting (opening) the tourniquet even at the pre-hospital stage?**

Yes

No

No information provided

Single-choice question*

31) **After applying a tourniquet and subsequently stabilising the patient, have you observed the bleeding to resume, even though it had been brought under control after the tourniquet was applied?**

Yes

No

No information provided

Single-choice question*

32) **After applying a tourniquet and carrying out further haemostatic measures (e.g. pressure bandages and haemostatic agents), did you attempt to release the tourniquet?**

Yes

No

No information provided

Single-choice question*

33) **Have you ever had to loosen a tourniquet, for example because the patient was feeling uncomfortable?**

Yes

No

No information provided

Single-choice question*

34) **Was the tourniquet you applied reopened in the trauma room?**

Yes

No

No information provided

Single-choice question*

35) **If so, have you noticed any bleeding since then?**

Yes

No

No information provided

Single-choice question*

36) **Have you ever had to apply a tourniquet to a child?**

Yes

No

No information provided

**Opinions**

Single-choice question*

37) **Do you feel confident, or would you feel confident, about using a tourniquet?**

Yes

No

No information provided

Single-choice question*

38) **Do you think applying a tourniquet is easy to learn?**

Yes

No

No information provided

Single-choice question*

39) **Do you find applying a tourniquet easy?**

Yes

No

No information provided

Single-choice question*

40) **Do you think applying a tourniquet is time-consuming?**

Yes

No

No information provided

Single-choice question*

41) **Do you think using a tourniquet is effective?**

Yes

No

No information provided

Single-choice question*

42) **Do you consider the use of a tourniquet to be low-risk?**

Yes

No

No information provided

Single-choice question*

43) **Would you be inclined to apply a tourniquet in a wide range of situations?**

Yes

No

No information provided

Single-choice question*

44) **Do you consider a tourniquet to be non-essential in the emergency services’ kit?**

Yes

No

No information provided

**Thank you very much for taking part!**

We would like to thank you very much for your active participation in this survey. If you are interested in the results of the survey, please send a brief email to michael.lautenschlaeger@bergmannstrost.de.

Kind regards,

Michael Lautenschläger
