# Supplementary material for: Tourniquet use in different emergency medicine professional groups, results from a regional cross-sectional online survey
Source: BMC Emerg Med. 2026 Aug 3;26:214. doi: 10.1186/s12873-026-01674-w (PMC13445843; doi:10.1186/s12873-026-01674-w)
Supplement: Supplementary file 2 [file 12873_2026_1674_MOESM2_ESM.docx]

***Supplemental Material***

***General overview of data already been published in a German-language journal.***

***Objective of the German paper****: A status survey of the level of training and frequency of use*

*of tourniquets in the context of current recommendations and guidelines.*

***Data source****: The same survey as described in the current paper with the same survey period but different number of participants due to the fact that only participants completing the whole survey were included.*

***Data overlapping:*** *132 respondents included in the German-language paper, table 1 has almost identical categories, but the results differ slightly due to the different numbers of participants.*

***Statistical methods:*** *Only descriptive statistic non-statistical testing for different between groups.*

***Results:*** *Short one-page* *description of the status survey mentioning the percentage of education, practical use and availability.*

***Discussion:*** *totally different*

***Supplemental Table 1)*** *Adherence to the existing German guidelines on tourniquet use (questions 17 to 19 of the survey).*

| *Question* | *Right answer(s)* | *Wrong answer(s)* | *Total answers* |
| --- | --- | --- | --- |
| *17) When would you apply a tourniquet?* | *540 (93%)* | *38 (7%)* | *578* |
| *18) Where would you apply a tourniquet?* | *126 (77%)* | *38 (23%)* | *164* |
| *19) How long would you estimate the max. duration of a tourniquet to be?* | *129 (92)* | *11 (8%)* | *140* |
| *Total number of answers* | *795 (90%)* | *87 (10%)* | *882 (100%)* |

*The table reflects proportion of guideline-concordant and not concordant answer options, not fully adherent individuals.*

*Questions 17 to 19 from the survey with each answer option and whether it was scored as correct*, incorrect**, or right but context-dependent***. Correct and right but context dependent answers were score as “right” answers in the table above.*

*Below one can find the above-mentioned questions and whether the answers are right, wrong or right but context dependent.*

17) ***When would you apply a tourniquet?***

*For injuries to limbs that can be compressed effectively. ***

*Regardless of the effectiveness of a pressure bandage. ***

*After applying a pressure bandage, but if bleeding persists. **

*For multiple injuries to a limb, with several sources of bleeding. **

*In the event of multiple casualties. ****

*For inaccessible limb injuries. ****

*For limb injuries close to the trunk. ***

*To stop bleeding whilst other actions are required, e.g. securing the airway at the same time*

*only as a last resort. ****

18) ***Where would you place a tourniquet?***

*Directly above the site of bleeding. ***

*As distally as possible on the limb, but sufficiently proximal to the source of bleeding (e.g. a hand’s width). **

*Directly over the bleeding wound (for better compression). ***

*Over a possible fracture (to stabilize the fracture). ***

*Over the joint of the affected limb. ***

19) ***How long do you estimate the maximum duration of a tourniquet to be?***

*30 min ****

*60 min ****

*90 min ****

*120 min **

*150 min ***

*180 min ***

*Unlimited ***

**Respondent-level composite outcome on guideline adherence:**

fully concordant on indication:	*93%*
fully concordant on location:	*77%*
fully concordant on occlusion time:	*92%*
concordant on all three domains:	77%
